# Raynaud's Phenomenon During Non-operating Room Anesthesia: A Case Report

**DOI:** 10.7759/cureus.32906

**Published:** 2022-12-24

**Authors:** Sony Sony, Shivam Shekhar, Beeraling N Walikar, Shiwali Shiwali

**Affiliations:** 1 Anaesthesiology, All India Institute of Medical Sciences, Rishikesh, IND

**Keywords:** pediatric saturation probe, low saturation, blue fingers, pulse oximeter, non-operating room anesthesia, raynaud's phenomenon

## Abstract

Non-operating room anesthesia challenges the anesthesiologist to deliver the same high-quality care as in the operating room. Amid the perplexity of the unfamiliar environment, scarcity of ancillary staff, and physical limitations, a distressing signal from pulse oximetry can cause a scare. We present a case of Raynaud’s phenomenon in a patient posted for cystogastrostomy in the endoscopic retrograde cholangiopancreatography suite. The patient had pulmonary complications, a left-sided pleural effusion with underlying lung collapse related to pancreatitis; thus, a non-reassuring reading from pulse oximetry caused alarm. The patient had sinus tachycardia, with a heart rate of 104 beats per minute, and a blood pressure of 100/60 mmHg. We provided supplemental oxygen to the patient while planning for emergency tracheal intubation because of a low peripheral oxygen saturation of 87%. The patient was conscious during this time, prompting us to check the pulse oximeter probe. We then noticed that patient's digits had turned blue/pale. A sudden attack of Raynaud's in the perioperative period can mislead the caregivers, and an unwarranted state of panic can ensue.

## Introduction

Raynaud's phenomenon (RP) is a relatively common but often unrecognized clinical syndrome, causing characteristic color changes in the digits, due to vasospasm [1]. Even though it is less encountered among anesthesiologists, yet, its recognition can prevent panic during perioperative care. We present a case of RP in a patient at high risk for desaturation for non-operating room anesthesia (NORA), which caused alarm in an already stressful situation. We obtained written informed consent from the patient for publication of the case.

## Case presentation

A 30-year-old male patient presented to our emergency department. He had features of acute pancreatitis with gross ascites and left-sided pleural effusion with underlying lung collapse. The patient gave a history of acute, alcohol-induced pancreatitis six months prior to the procedure. He received treatment at the local hospital at that time. He presented to our emergency department with a chief complaint of acute abdomen. His vitals were stable, with a heart rate of 90 beats per minute, blood pressure of 100/60 mmHg, and oxygen saturation (SpO2) of 96% on room air. Mild pallor, pedal edema, and moderate ascites were present, but without icterus or cyanosis. On auscultation, we heard fine crepitus on the left lung base. On physical examination, we could not detect any other abnormalities. His laboratory investigations were within normal limits except for hemoglobin of 9.5 g/dl and a positive antinuclear antibody titer of 1:100. High-resolution computed tomography (HRCT) of the chest revealed left-sided pleural effusion with underlying lung collapse. Magnetic resonance cholangiopancreatography (MRCP) showed features of acute pancreatitis with gross ascites and collection in the lesser sac showing suspicious communication with the main pancreatic duct. The surgical team did a diagnostic pleural tap and a diagnostic ascitic tap. The pleural fluid showed elevated lipase of 6370 U/L, amylase of 5200 U/L, and lactate dehydrogenase of 287 U/L. The peritoneal fluid had raised protein of 36 g/L and was chylous in appearance. Next, he was posted for endoscopic retrograde cholangiopancreatography (ERCP) and cystogastrostomy under general anesthesia. The pulmonology team assigned a high-risk category for anesthesia. The anesthesia team did a pre-anesthetic evaluation. His weight was 45 kg and his height was 170 cm. He gave a history of alcohol use disorder and a loss of 2 kg of weight in the past six months. The detailed medical history and examination did not reveal any additional information. As per institutional protocol, we asked him to be nil per oral for eight hours for solid food and two hours for water.

**Figure 1:**
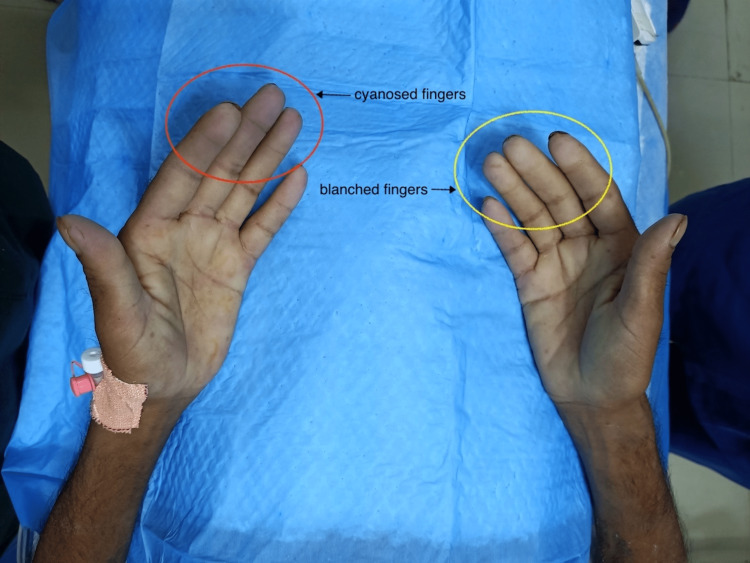
The bluish/cyanosed fingers (red circle) of the left hand and blanched fingers (yellow circle) of the right hand of the patient

On the day of the procedure, we gave him a tablet of ranitidine 150 milligrams before transporting him to the ERCP suite. All the necessary arrangements for the conduct of NORA were made by the anesthesia team. In an anticipation of raised intra-abdominal pressure, we planned a rapid sequence induction of anesthesia. Upon the arrival of the patient in the ERCP suite, we connected all the standard American Society of Anesthesiologists (ASA) monitoring devices to him. His heart rate was 104 beats per minute with a blood pressure of 100/80 mmHg. To our surprise, the SpO2 reading showed 87% saturation, which caused alarm. We provided supplemental oxygen to the patient through an already-connected anesthesia circuit while discussing a plan for emergency intubation. The patient was conscious, which prompted the evaluation of the pulse oximetry probe. While doing so, we noticed the blanched and cyanosed fingers of the patient (Figure 1).

**Figure 2:**
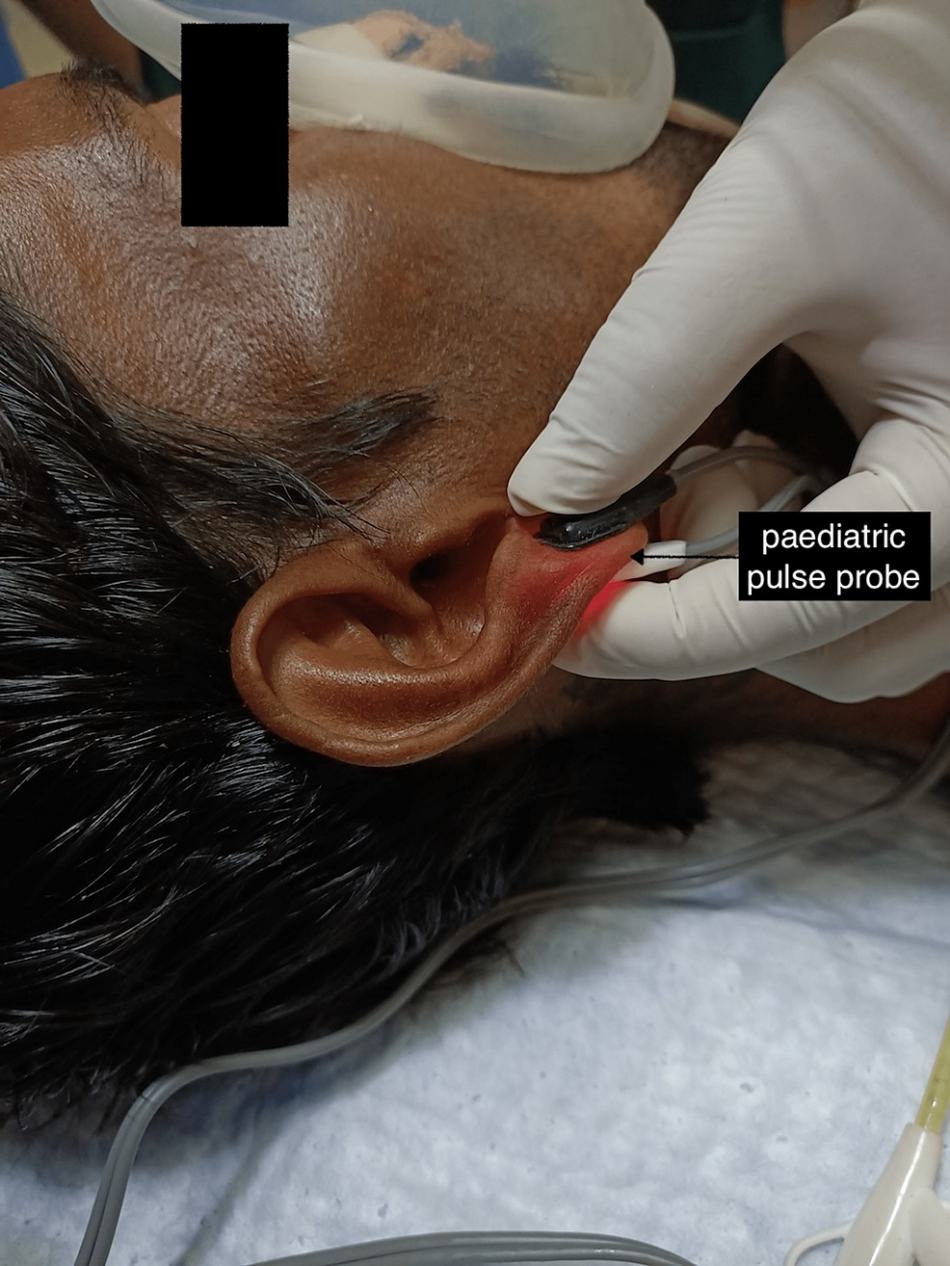
Pediatric pulse oximeter probe placed on the ear lobule of the adult patient

We did not get any different result by putting the SpO2 probe on his toes. When we asked him, the patient confirmed that sometimes in cold, his fingers would turn blue. Although the ambient temperature of the ERCP suite was normal, the patient's extremities were cold. Even after warming his hands with warm fluid bottles, the waveforms on the peripheral pulse probe did not appear and the numerical value fluctuated between 70% and 85%. In the ERCP suite, we did not have an adult ear lobe SpO2 probe but we did have a pediatric one. We then attached a pediatric ear lobe probe to his left ear lobule (Figure 2).

This time we could get a waveform with a reading of 98% saturation. Due to fear of any accidental localized vasospasm, we abandoned the plan to use thiopentone. We proceeded with fentanyl 90 micrograms for analgesia and propofol 90 milligrams followed by succinylcholine 50 milligrams for rapid sequence induction of anesthesia. During this time, we continued oxygenation while also giving a cricoid pressure. We used an 8.0-millimeter internal diameter tracheal tube to secure the patient's airway and maintained the anesthesia with an air-oxygen mixture with sevoflurane 2%. We used warm intravenous fluid during the procedure and covered the patient with extra gowns to maintain his temperature at around 36-37°C. There were no significant hemodynamic changes during the 60 minutes procedure. Postoperatively, we observed him in the recovery area for one hour, followed by a transfer to the ward. We advised the patient to reveal RP in his future medical history.

## Discussion

RP is classically described with a triphasic color change of the digits with initial white or pallor (ischemic phase), then blue or cyanosis (deoxygenation phase), followed by red or erythema (reperfusion phase) [2]. Its diagnosis is clinical. Patients give a history of unusual sensitivity to cold, with fingers changing color, often turning blue/white or both on exposure to cold. There is an imbalance between vasoconstriction and vasodilation, affected by local and neurohumoral factors. It is typically present in the hands but can also affect the toes, nose, earlobes, or nipples. Other than cold, reported triggers include emotional stress, medications, injury, smoking, and the presence of other arterial diseases [3]. Normal antinuclear antibody (ANA) level is no longer a requisite for the diagnosis of primary Raynaud's but it may be helpful or diagnostic for secondary Raynaud’s [4]. The underlying cause could be of clinical significance to the treating physician and the anesthesia team.

In retrospect, our patient gave a positive history to make a clinical diagnosis of RP. Albeit this was upon asking leading questions. No attacks of RP happened during his two-day stay at our hospital, before ERCP. The ANA titer was positive in our patient, which could have prompted us for possible connective tissue disorder and better anesthetic preparedness. This particular attack involved all his digits, which resulted in misleading information. Luckily, we could obtain a reading from the ear lobe, and the plethysmographic waveform assured us of its reliability. The peripheral pulse oximeter uses an algorithm looking for arterial pulsations. In patients with low perfusion due to hypotension, cold extremities, or Raynaud's disease, pulse oximetry signals can be unreliable or even difficult to obtain. Other application sites such as earlobes and forehead, nasal alar, or lip have been successfully used [5]. The conduct of NORA has its limitations. These peripheral suites are designed specifically for the procedure being performed there. The considerations for anesthesia-related concerns are limited. The ancillary staff is not always a trained anesthesia technician/nurse, thus a low threshold for consternation.

Raynaud's disease is fairly common and affects 3-5% of the global population [6,7]. These patients presenting for surgeries may pose unique challenges to the anesthesia team. Any stimuli or stress during the perioperative period can lead to an attack of RP. Often the operation theatres are cold. The use of warm intravenous fluid and keeping operating theatres warm can prevent the precipitation of RP. In these patients, we should avoid vasoconstricting agents like beta-blockers, phenylephrine, mephentermine, epinephrine, norepinephrine, diazepam, thiopental, and phenytoin. Pulse oximetry is a standard-of-care device. Any distressing reading from it can lead to unnecessary investigations and interventions like arterial blood gas sampling and oxygen supplementation. Arterial cannulation of distal extremities can worsen the pre-existing condition, so we should avoid it.

## Conclusions

Anesthesiologists do not routinely encounter RP. A simple leading question during pre-anesthetic evaluation, such as do your fingers change color on exposure to cold, may perhaps help us decrease morbidities in patients of RP, should we encounter them. Alertness toward the attack in the perioperative period can further prevent unwarranted panic.
